# Enrichment of Pluripotent Stem Cell-Derived Hepatocyte-Like Cells by Ammonia Treatment

**DOI:** 10.1371/journal.pone.0162693

**Published:** 2016-09-15

**Authors:** Daihachiro Tomotsune, Kanji Hirashima, Masako Fujii, Fengming Yue, Ken Matsumoto, Sakiko Takizawa-Shirasawa, Tadayuki Yokoyama, Katsunori Sasaki

**Affiliations:** 1 Department of Biotechnology and Biomedical Engineering, Institute for Biomedical Sciences, Interdisciplinary Cluster for Cutting Edge Research, Shinshu University Matsumoto, 3-1-1 Asahi, Matsumoto 390–8621, Japan; 2 Department of Histology and Embryology, Shinshu University School of Medicine, 3-1-1 Asahi, Matsumoto 390–8621, Japan; 3 Nissui Pharmaceutical Co., Ltd., 1075–2 Hokunanmoro, Yuki, Ibaraki 307–0036, Japan; 4 Bourbon Corporation, 4-2-14 Matsunami, Kashiwazaki, Niigata 945–8611, Japan; National Cancer Institute, UNITED STATES

## Abstract

Embryonic stem cells (ESCs) and induced pluripotent stem cells (iPSCs) are potential resources for the regeneration of defective organs, including the liver. However, some obstacles must be overcome before this becomes reality. Undifferentiated cells that remain following differentiation have teratoma-forming potential. Additionally, practical applications require a large quantity of differentiated cells, so the differentiation process must be economical. Here we describe a DNA microarray-based global analysis of the gene expression profiles of differentiating human pluripotent stem cells. We identified differences and commonalities among six human pluripotent stem cell lines: the hESCs KhES1, KhES2, KhES3, and H1, and the iPSCs 201B7 and 243G1. Embryoid bodies (EBs) formed without requiring supplementation with inducing factors. EBs also expressed some liver-specific metabolic genes including the ammonia-metabolizing enzymes glutamine synthetase and carbamoyl-phosphate synthase 1. Real-time PCR analysis revealed hepatocyte-like differentiation of EBs treated with ammonia in Lanford medium. Analysis of DNA microarray data suggested that hepatocyte-like cells were the most abundant population in ammonia-treated cells. Furthermore, expression levels of undifferentiated pluripotent stem cell markers were drastically reduced, suggesting a reduced teratoma-forming capacity. These results indicate that treatment of EBs with ammonia in Lanford medium may be an effective inducer of hepatic differentiation in absence of expensive inducing factors.

## Introduction

Cell replacement therapies using hepatocytes generated in vitro may be useful in treating fatal liver disease [1,2]. Pluripotent stem cells, such as embryonic stem cells (ESCs) and induced pluripotent stem cells (iPSCs), are promising resources for cell therapy because they can expand continuously in an undifferentiated state and can produce any type of tissue given appropriate conditions [3,4].

Multiple reports describe successful induction of hepatic differentiation from both ESCs and iPSCs [5,6]. However, several problems remain unresolved. Undifferentiated pluripotent stem cells can form teratomas following host transplantation [7]. Additionally, hepatic differentiation of pluripotent cells is not complete, with various cell types—including undifferentiated pluripotent stem cells—remaining in the induced cell population [8]. Furthermore, practical applications of cell replacement therapy require a huge number of hepatocytes. Therefore, the cost of hepatocyte production must be low and a requirement for expensive inducing factors such as activin and FGFs is undesirable.

The liver is the central site of drug metabolism and multiple liver-specific metabolic pathways operate here. Tomizawa et al. [9,10] exploited these liver-specific pathways to develop a selective medium for hepatocyte culture. This medium contains galactose and ornithine, lacks glucose and arginine, facilitates culture of healthy primary human hepatocytes, and eliminates undifferentiated human iPS cells. Furthermore, Kondo et al. [11] selected differentiated hepatocyte-like cells from human iPSCs using similar media. However, these protocols require an expensive induction process which utilizes activin to promote hepatocyte differentiation. The same is true of other strategies reported for the isolation of pluripotent stem cell-derived hepatic cells [12,13].

We performed a global gene expression analysis of six differentiating pluripotent stem cell lines and identified several hepatocyte-specific genes that are expressed at the early induction stage of hepatic differentiation in the absence of expensive inducing factors. Carbamoyl-phosphate synthase 1 (CPS1) and glutamine synthetase (Glul)—which catalyze and eliminate ammonia—were two such genes. Ammonia is well known to affect cultured cells, reducing growth rate and inducing cell death [14]. This suggests that ammonia could select for and enrich populations of pluripotent stem cell-derived hepatocytes. Therefore, we included ammonia in our hepatocyte induction protocol and again analyzed global gene expression using DNA microarray.

## Materials and Methods

### Pluripotent stem cells and culture

This study was approved by the Shinshu University Institutional Review Board in accordance with *The Guidelines for Derivation and Utilization of Human Embryonic Stem Cells* by The Ministry of Education, Culture, Sports, Science, and Technology of Japan. Pluripotent stem cell lines used in this study are listed in Table 1. The evaluations of Table 1 were based on our experience [12, 15–23]. The H1 hESC line was purchased from WiCell Research Institute (Madison, WI, USA). Three KhES cell lines were supplied from the Institute for Frontier Medical Science (Kyoto University, Kyoto, Japan). Two human iPS cell line, 253G1 and 201B7, were supplied from RIKEN Bio Resource Center (Tsukuba, Ibaraki, Japan).

**Table 1:** Features of 6 pluripotent stem cells in cell handling.

cell line	hESC or iPSC	Maintenance	Tendency of differentiation	survival after thawing
KhES1	hESC line	easy	weak	good
KhES2	hESC line	easy	weak	poor
KhES3	hESC line	moderate	marked	good
H1	hESC line	difficult	marked	moderate
253G1	hESC line	easy	moderate	good
201B7	hESC line	easy	weak	good

Pluripotent cells were cultured on a feeder cell layer of mouse embryonic fibroblasts (MEFs; Oriental Yeast Co, Tokyo, Japan) inactivated with mitomycin C (Kyowa Hakko Kirin Co, Tokyo, Japan). The culture medium consisted of 80% KnockOut™ Dulbecco’s minimal Eagle’s medium (DMEM; Gibco, Grand Island, NY, USA) supplemented with 20% KnockOut™ Serum Replacement (Gibco), 100 μM non-essential amino acids (Wako, Chuo-ku, Osaka, Japan), 2 mM L-glutamine (Wako), 100 μM 2-mercaptoethanol (Sigma, St. Louis, MO, USA), and 4 ng/mL basic fibroblast growth factor (Wako). For feeder-free culture, contaminating MEFs were removed by incubating the cell suspension on a gelatin-coated plate at 37°C for 2 h in maintenance culture medium. Here, MEFs adhered to the plate but hESCs did not [24]. Cells remaining in suspension were transferred to a fresh dish and cultured on Matrigel (BD Biosciences, San Jose, CA)-coated plate in MEF-conditioned ES medium or Essential 8 medium (Thermo Fisher Scientific, Waltham, MA).

#### EB formation

hESCs were disbursed into small clumps (200 μm diameter) by treatment with 0.25% trypsin and 0.1 mg/mL collagenase IV (Invitrogen, Carlsbad, CA) in phosphate-buffered saline (PBS) containing 20% Knockout™ Serum Replacement and 1 mM CaCl2 at 37°C for 5 min, followed by pipetting. Clumps of 2.5–3 × 105 cells were transferred to a non-adhesive 60-mm dish coated with Lipidure (NOF Corporation, Tokyo, Japan). Cells were cultured in 4 mL of EB medium: 80% knockout DMEM, 100 μM non-essential amino acids, 2 mM L-glutamine, 100 μM 2-mercaptoethanol, and 20% fetal bovine serum (Hyclone, Logan, UT, USA). Suspension cultures were maintained for 5 days.

#### Ammonia induction

Sterile distilled water was added to MEF-coated dishes to remove MEFs but retain the cell adhesion capability of the plate surface. Water was aspirated along with cellular debris at least 1 h later. Pluripotent stem cells prepared using the standard cell passage method were added to the treated dish in ES medium and cultured in 3%-CO2 incubator at 37°C for 48 h. ES medium was exchanged with EB medium the next day. At approximately 70% cell confluency, 10 μL/mL of dimethyl sulfoxide (DMSO; Sigma–Aldrich, St. Louis, MO, USA) was added to dishes in fresh EB medium. This process was repeated twice. DMSO-treated cells were separated using a cell scraper, transferred into a non-adhesive dish (Lipidure-coated plate, NOF Corporation), and cultured in Lanford medium [16, 25–27] for 2 days. Following EB formation, 1 μL/mL of 10% w/v aqueous ammonia (Wako) was added to fresh Lanford medium. This process was repeated every 2 days for 14 days total.

#### Quantitative real-time RT-PCR

Total RNA was extracted using TRIzol reagent (Invitrogen) according to the manufacturer’s instructions. Quantitative PCR analysis was performed as described previously [17] using the Thermal Cycler Dice Real-Time System (Takara Bio, Otsu, Japan). Primer sequences for *Nanog*, *Oct3/4*, *NCAM*, *brachyury (T)*, *goosecoid (GSC)*, *Sox17*, *Sox7*, *HNF3b*, *HNF1A*, *HNF4A*, *alpha-fetoprotein (AFP)*, *albumin (ALB)*, *transthyretin (TTR)*, *CYP3A7*, *TDO2*, *UGT2B7*, *ASGR1*, *OTC*, *SAA1*, *GLUL*, *CPS1*, *PCNA*, *b-actin* are provided in (Table 2).

**Table 2:** Genes, primers, and functions.

Gene	Primer sequences	Function
*Nanog*	TTACCGCGGCAAGAACAT	pluripotentstemcellmarker
	CCACCTGCAGAGAAACTGC	
*Oct3/4*	TCTATTTGGGAAGGTATTCAGC	pluripotentstemcellmarker
	ATTGTTGTCAGCTTCCTCCA	
*NCAM*	TTACCGCGGCAAGAACAT	Neuralcellmarker
	CCACCTGCAGAGAAACTGC	
*brachyury (T)*	TGCTTCCCTGAGACCCAGTT	Mesodermmarker
	GATCACTTCTTTCCTTTGCATCAAG	
*Sox17*	GGCGCAGCAGAATCCAGA	Endodermmarker
	CCACGACTTGCCCAGCAT	
Sox7	ACGCCGAGCTCAGCAAGAT	Endodermmarker
	TCCACGTACGGCCTCTTCTG	
*HNF3b*	GGGAGCGGTGAAGATGGA	Hepaticprogenitormarker
	TCATGTTGCTCACGGAGGAGTA	
*HNF1A*	GTGACCCAGAGCCCCTTC	Hepaticprogenitormarker
	GGGCTTGTGGCTGTAGAGG	
*HNF4A*	CATGGCCAAGATTGACAACCT	Hepaticprogenitormarker
	TTCCCATATGTTCCTGCATCAG	
*AFP*	TGCAGCCAAAGTGAAGAGGGAAGA	Endoderm/hepatocytemarker
	CATAGCGAGCAGCCCAAAGAAGAA	
*ALB*	TGCTTGAATGTGCTGATGACAGGG	Hepatocytemarker
	AAGGCAAGTCAGCAGGCATCTCATC	
*TTR*	GCCGTGCATGTGTTCAGA	Hepatocytemarker
	GCTCTCCAGACTCACTGGTTTT	
*CYP3A7*	GGAACCCGTACACATGGACT	Hepatocytemarker
	CATGTCAAACGTCCAATAGCC	
*TDO2*	GGAGAAGAAAATGAACTGCTACTTAAA	Hepatocytemarker
	GGCTCTAAACCTGGAGTTCTTTC	
*UGT2B7*	TGGATACCCCAGAATGACCT	Hepatocytemarker
	GATCGGCAAACAATGGAATC	
*ASGR1*	TGGATACCCCAGAATGACCT	Hepatocytemarker
	CGCAGGTCAGACACGAACT	
*OTC*	TTTCCAAGGTTACCAGGTTACAA	Hepatocytemarker
	CTGGGCAAGCAGTGTAAAAAT	
*SAA1*	GGGCATACAGCCATACCATT	Hepatocytemarker
	ACCAAGGAGCAGAAAACCAG	
*GLUL*	TCTCGCGGCCTAGCTTTA	Ammoniametabolism
	CATTCGTTGCTTTCTTCGTG	
*CPS1*	CAAGTTTTGCAGTGGAATCG	Ammoniametabolism
	ACTGGGTAGCCAATGGTGTC	
*PCNA*	TGGAGAACTTGGAAATGGAAA	proliferatingcellmaker
	GAACTGGTTCATTCATCTCTATGG	
*b-actin*	TGGCACCCAGCACAATGAA	Housekeepinggene
	CTAAGTCATAGTCCGCCTAGAAGCA	

The comparative threshold cycle method was used to analyze data, with gene expression levels assessed relative to that of the housekeeping gene *β-actin*. At least three independent experiments were performed for each condition and data are expressed as means ± standard deviation (SD). Values were compared using the Student’s *t*-test, with a *P-*value < 0.05 considered statistically significant.

#### DNA microarray

Total RNA was amplified and labeled with Cy3 using Agilent Low Input Quick Amp Labeling Kit, one-color (Agilent Technologies, Palo Alto, CA). Total RNA (100 ng) was transcribed to double-strand cDNA using a poly dT-T7 primer and cDNA products were then used as templates for *in vitro* transcription to generate fluorescent cRNA. For each hybridization reaction, 1.65 μg of fragmented Cy3-labeled cRNA was incubated with an Agilent Human GE 8x60K Microarray (Design ID: 028004) at 65°C for 17 h. Washed microarrays were scanned using an Agilent DNA microarray scanner.

#### Data analysis of microarray

Intensity values of each scanned feature were quantified using Agilent feature extraction software version 10.7.3.1. Only features which were flagged as no errors (Detected flags) were used. Features which were not positive, not significant, not uniform, not above background, saturated, or population outliers (Compromised and Not Detected flags) were excluded. The data were normalized using Agilent GeneSpring GX version 13.1.1 (per chip: normalization to 75^**th**^ percentile shift; per gene: normalization to median of all samples). There are 42,405 probes on each microarray chip, without including control probes. We required ≥ 2-fold- change in signal intensity to classify a change as significant.

#### FMatch analysis

BIOBASE TRANSFAC (Qiagen, Valencia, CA, USA) was used to examine the −600 to +50 region of genomic sequence surrounding the transcription start sites of changed and unchanged genes for potential transcription factor binding sites. Parameters employed were: Matrix library: TRANSFAC MATRIX TABLE, Release 2015.3, Profile: vertebrate_non_redundant_minSUM (Only high-quality matrices), P-value threshold: 0.01.

#### Western blot analysis

Whole cell lysates were prepared and subjected to western blot analysis with monoclonal antibody specific for human albumin (Clone hAlb 3-7A, Takara Bio, Otsu, Japan). Antibody for b-actin was used as a control.

#### Immunostaining of cultured cells

Ammonia-treated EBs at day 12 were transferred to matrigel-coated glass cover slips and incubated in Lanford medium with ammonia for 2 days. The attached EBs were fixed in 4% paraformaldehyde in 0.1 M phosphate buffer, pH 7.4, for 1 h at room temperature and immunostained as described previously [23]. Antibodies used were as follows: ALB (Clone hAlb 3-7A, Takara Bio, Otsu, Japan), AFP (goat polyclonal; Dako).

#### CYP3A4/7 activity assay

CYP3A4/7 activity in 1 ug (protein quantity) of microsome fraction extracted from undifferentiated cells or of ammonia-treated EB was analyzed using a p450-Glo CYP3A4 Assay kit with Luciferin-BE (Promega, Madison, WI) in accordance with the manufacturer’s instructions. The values of results were fold-change in light units relative to the value of undifferentiated cells.

## Results

### Cell line-specific differences and commonalities in pluripotent cell differentiation

**Fig 1:**
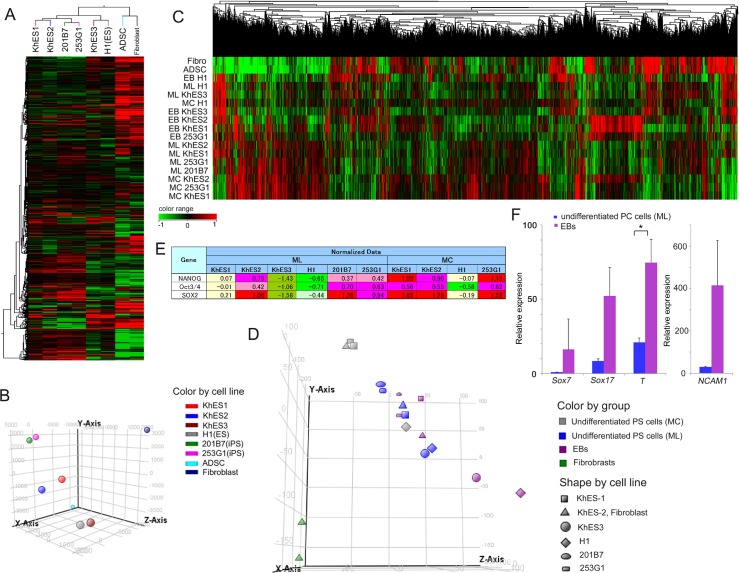
Microarray data indicating the differentiation tendencies of six pluripotent stem cell lines. Heat maps of DNA microarray analysis (A, C) and the associated principal component analysis (B, D) of undifferentiated cells (A, B) and EB cells (C, D). Hierarchical Clustering; Similarity Measure: Euclidean; Linkage Rule: Complete. (E) Clipped representation from normalized microarray data showing pluripotent stem cell marker genes. (F) Quantitative PCR analysis of expression of endoderm markers Sox7 and Sox17, mesoderm marker Brachyury (T), and neuroectoderm marker NCAM1 in 253G1 cells. Each bar represents mean and SD (n = 3). Values are fold-change relative to the value of Sox7 in undifferentiated cells. *P < 0.05 compared with the value for undifferentiated cells. ML: undifferentiated maintenance culture on a MEF layer; MC: undifferentiated maintenance culture in MEF-conditioned medium on Matrigel; EB: embryoid bodies formed in static suspension culture.

We conducted DNA microarray analysis of total RNA samples from six pluripotent stem cell lines (four ESC and two iPSC lines listed in Table 1). Samples from cells in an undifferentiated state were obtained by conventional ES culture on a MEF layer (ML). Heat map representation and principal component analysis (PCA) revealed differences in the expression profiles of all six pluripotent stem cell lines in undifferentiated culture (Fig 1A and 1B). Inclusion of microarray data from pluripotent stem cell lines grown in a Matrigel-coated dish with MEF-conditioned medium (MCM) and under EB-forming conditions in the analysis revealed that differences in gene expression profiles arose during EB formation (Fig 1C and 1D). Pluripotent stem cells cultured in MCM maintained an undifferentiated state than those grown on a ML (Fig 1E). EB formation can induce spontaneous differentiation of pluripotent stem cells. Consistently, we detected markers of all three germ layers in EBs (Fig 1F). Our findings reveal that the six undifferentiated pluripotent stem cell lines have a varied differentiation tendency.

### Pluripotent stem cell EBs express ammonia-metabolizing enzymes

**Fig 2:**
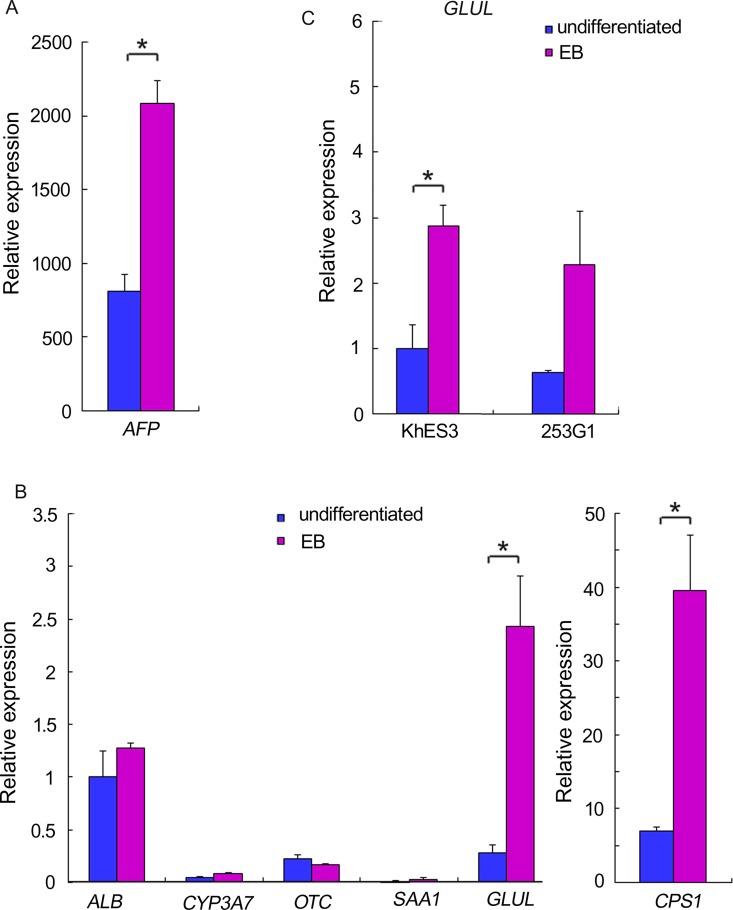
Expression of hepatocyte makers in EBs. Graphs depict quantitative PCR analysis of *AFP* (A), *albumin (ALB)*, *CYP3A7*, *OTC*, *SAA1*, *CPS1* (B) and *GLUL* (B, C) gene expression in H1 ES cells (A, B), KhES3 ES cells and 253G1 iPS cells (B). Each bar represents mean and SD (n = 3). Values are fold-change relative to the value for *ALB* in undifferentiated H1 cells (A, B) or undifferentiated KhES3 cells (C). *P < 0.05 compared with values for undifferentiated cells.

DNA microarray analysis suggested that EB formation was unable to induce pluripotent stem cells to differentiate into hepatocytes. While the hepatocyte marker *AFP* was upregulated in EBs (Fig 2A), other markers of mature hepatocytes (*ALB*, *CYP3A7*, *OTC*, and *SAA1*) were either not expressed in EBs or were not upregulated during the differentiation process (Fig 2B). However, the ammonia-metabolizing enzymes *GLUL* and *CPS1* were both upregulated in EBs (Fig 2B and 2C). Therefore, including ammonia in the culture medium of differentiating pluripotent cells may promote selection of immature hepatocytes.

### DNA microarray analysis of ammonia-treated cells reveals a common direction to hepatocyte-like differentiation

**Fig 3:**
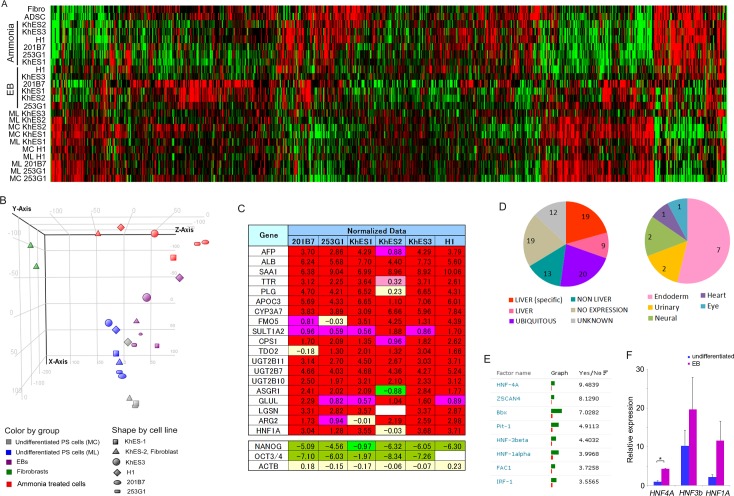
DNA microarray data reveals hepatocyte-like differentiation of ammonia-treated cells. Results of DNA microarray analyses were combined and are presented as a heat map (A) and a principal component analysis (B). (C) Clipped representation from normalized microarray data showing hepatocyte related genes (upper) and control genes (lower). (D) Cell-type specificity of highly-expressed genes in ammonia-treated cells. Cell-type was annotated using GENATLAS (Paris Descartes University; http://genatlas.medecine.univ-paris5.fr/google/gene.php#). Left; “LIVER” indicates gene with high or predominant expression in organ(s) including the liver. “LIVER (specific)” indicates gene with high liver specific expression. “NON LIVER” indicates genes with high expression levels in organs other than the liver. “NO EXPRESSION” indicates genes without high expression. “UBIQUITOUS” indicates genes with ubiquitous expression (including liver expression). “UNKNOWN” indicates genes with no expression data. Right; breakdown of “NON LIVER”. (E) The top eight factors returned from TRANSFAC analysis of ammonia-treated cells. (F) Quantitative PCR analysis of *HNF4A*, *HNF-3beta* (*HNF3b*), and *HNF-1alpha* (*HNF1A*), gene expression levels in H1 ES cells. Each bar represents mean and SD (n = 3). Values are fold-change relative to the value of *HNF4A* in undifferentiated cells. *P < 0.05 compared with the value for undifferentiated cells. ML: undifferentiated maintenance culture on MEF layer; MC: undifferentiated maintenance culture in MEF-conditioned medium on Matrigel; EB: embryoid bodies formed in static suspension culture.

We treated EBs from six pluripotent stem cell lines with ammonia in Lanford medium for 14 days. DNA microarray analysis showed that ammonia treatment altered the gene expression profile in EBs from each pluripotent stem cell line (Fig 3A). PCA of total microarray data suggested progression of differentiation (Fig 3B). Additionally, many hepatocyte markers were significantly upregulated in ammonia-treated cells (Fig 3C).

We next sorted microarray probe data based on the expression scores (normalized data) of KhES1 cells, as PCA of data from these cells revealed ordinary and mild changes in gene expression following ammonia treatment. We then isolated the top 200 probes (genes) that had an altered expression level, and further selected 92 non-overlapping genes for which at least five of the six cell lines returned positive scores (S1 Table). The cell-type specificity of these genes is indicated in Fig 3D, suggesting that hepatocyte-like cells were a major cell type present following ammonia treatment.

We next explored potential upstream factors involved in ammonia-mediated differentiation. We conducted an FMatch analysis with the TRANSFAC database using the 313 genes selected as described above and 120 additional unchanged genes as controls. The hepatic transcription factors *HNF4A*, *HNF-3beta* (*HNF3b*), and *HNF-1alpha* (*HNF1A*) were identified in the top six results returned (Fig 3E). Furthermore, these hepatic transcription factors were also expressed in EBs (Fig 3F). Taken together, these findings suggest that hepatic differentiation occurred in response to ammonia treatment of pluripotent cells.

### Confirmation of hepatocyte-like differentiation in ammonia-treated cells

**Fig 4:**
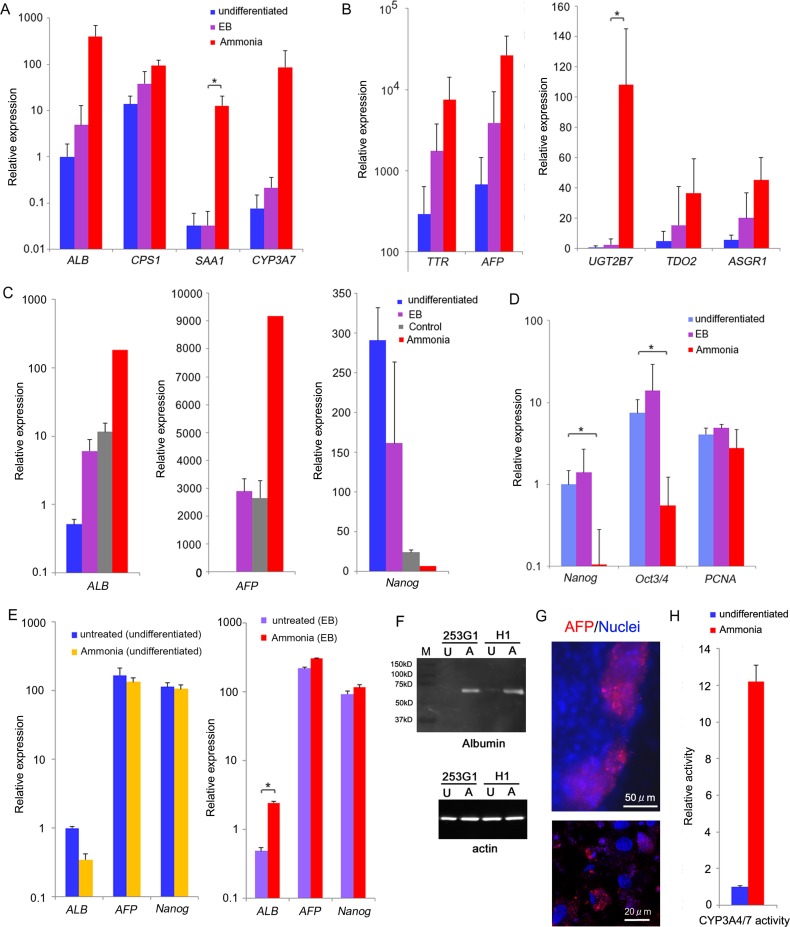
Characterization of ammonia-induced hepatocyte-like cells. (A, B) Expression of hepatocyte makers in undifferentiated, EB, and ammonia-treated cells. (C) Expression levels of *ALB*, *AFP*, and *Nanog* gene in undifferentiated, EB, and control (DMSO treated EBs cultured 14 days in Lanford medium without ammonia) 253G1 cells. (D) Expression levels of pluripotent stem cell makers (*Nanog* and *Oct3/4*) and the proliferating cell maker *PCNA* in undifferentiated, EB, and ammonia-treated cells. (E) Expression levels of ALB, AFP, and Nanog gene in ammonia-treated undifferentiated H1 cells and H1 EBs (pre-cultured for 2 days). (F) Western blot analysis of ALB protein expression in undifferentiated and ammonia-treated cells. M: Marker proteins (pre-stained); U: undifferentiated cells; A: ammonia-treated cells. (G) Immunostaining of AFP in ammonia-treated H1 cells. (H) CYP3A4/7 activity of 253G1 cells assessed using assay kit. Each bar in A, B, and D represents the mean expression level in four pluripotent stem cell lines: KhES1, KhES3, H1, and 253G1 (n = 4) with SD. The values of A, B, and C are fold-change relative to the values for *ALB* in undifferentiated cells of A. The values of D are fold-change relative to the values for Nanog in undifferentiated cells. Each bar of undifferentiated, EB, and control in C represents mean and SD (n = 3). Each bar of ammonia-treated cells in C represents a single 253G1 cell sample (for reference). Each bar of undifferentiated and ammonia-treated cells in H represents mean and SD (n = 3). The values in H are fold-change relative to the values in undifferentiated cells. *P < 0.05 compared with the value for undifferentiated cells.

We used real-time PCR to analyze gene expression of several hepatic markers and confirmed the upregulation identified by DNA microarray (Fig 4A–4C). Only a small population of hepatocyte-like cells seemed to exist at the EB stage. However, we detected expression profile of many hepatocyte-like cells following ammonia treatment. This suggests that ammonia-metabolizing cell had enhanced proliferation abilities. High expression of *PCNA* in these cells supports this idea (Fig 4D). Additionally, markers of undifferentiated pluripotent stem cells were significantly decreased in ammonia-treated cells (Fig 4D), suggesting a reduced capacity for these cells to form teratomas.

To assess sensitivity of ammonia, we treated undifferentiated and EB-formed pluripotent stem cells with ammonia for 2days (Fig 4E). Real-time PCR analysis showed expression of most markers were not affected, however *ALB* in EB was slightly upregulated, suggesting that the effect of ammonia was specific to differentiated EB cultured in Lanford medium.

Finally, we confirmed hepatocyte-like cell differentiation at protein level. We found expression of ALB and APF in ammonia-treated cells by western blotting (Fig 4F) and immunostaining (Fig 4G) respectively. Based on the induction of CYP3A7 mRNA expression, we measured CYP3A4/7 activity and detected increased activity of ammonia-treated cells (Fig 4H).

## Discussion

We have demonstrated the enrichment of hepatocyte-like cells in EB cultures of various pluripotent stem cell lines following ammonia treatment. The ammonia detoxifying system is believed important for the evolution of tetrapods with cleidoic eggs [28,29]. Ammonia, a product of nitrogen metabolism, has strong neurotoxicity and must be rapidly eliminated from the embryonic development. Therefore, animals evolved from tetrapods may be equipped with an ammonia detoxifying system early in development. This notion is supported by our present results and also temporal analysis of *GLUL* and *CPS1* gene expression [30–33].

A toxic metabolite-based selection strategy similar to that used here has been employed to derive cardiomyocytes from pluripotent stem cells in the presence of lactate [34]. Although lactate and ammonia are two major metabolites with potential toxicity for cultured cells [35], lactate can be used as a selective energy source in glucose-depleted medium. Our protocol combines ammonia with Lanford medium which is a serum-free maintenance medium for hepatocytes [25]. Therefore, the potential synergistic effect of ammonia and Lanford medium may promote hepatocyte differentiation.

The hepatocyte-differentiation protocol presented here is simple and remarkably inexpensive because it does not require inducing factors. While multiple reports describe hepatic differentiation, to the best of our knowledge the level of hepatocyte enrichment we observed has not been previously achieved without an inducing factor. As practical applications of cell replacement therapy and large-scale drug testing require a huge number of differentiated cells, cell preparation protocols must be economical. Therefore, optimization of this ammonia-based protocol may render it useful in future cell replacement therapy applications.

## Supporting Information

S1 Table Sorted gene list for Fig 3D. (XLSX)
